# Telehealth in long-term care facilities during the Covid-19 pandemic – Lessons learned from patients, physicians, nurses and healthcare workers

**DOI:** 10.1186/s44247-022-00003-y

**Published:** 2023-01-24

**Authors:** Zhaoli Dai

**Affiliations:** 1 College of Medicine and Public Health, Flinders University, Sturt Road, Room 2.13 Health Sciences Building, Bedford Park, SA 5042 Australia; 2 School of Population Health, University of New South Wales, Sydney, NSW Australia

**Keywords:** Telehealth, Telemedicine, Long-term care facilities

## Abstract

**Background:**

Telehealth and telecare are particularly important and beneficial to long-term care facilities due to care demands, workforce, and the unique environment. Stemming from the recent findings on telehealth utilisation in residential aged-care facilities in Australia, this commentary seeks to identify lessons and perspectives learned during the Covid-19 pandemic from multiple users, including patients, physicians, nurses, and healthcare workers in long-term care (LTC) settings.

**Main body:**

From patients’ perspectives, older adults residing in LTC settings often opt not to use virtual care, with the majority preferring in-person visits. This is despite residents expressing their willingness to use telehealth, and virtual care has advantages in LTC settings or in remote areas. Additionally, hearing, vision, or cognitive impairment can limit residents’ ability to use information technology to access care, so their preferences for phone or video consultations depend on the health conditions or care requirement. From physicians’ perspectives, most healthcare practitioners have a positive attitude toward using telehealth. However, telephone consultations tended to be the dominant mode during the early period of the Covid-19 pandemic. Physicians also raised several major concerns, including technical and equipment-related issues, expanded roles, or additional workloads of LTC staff that could negatively affect clinical decision-making and unequal access in rural, older, and cognitively impaired patients. Most nurses and healthcare workers perceived telehealth positively as a way to enhance patients’ care access. However, the majority had concerns about acquiring appropriate knowledge of using the technology for themselves and their patients. In remote areas, nurses expressed higher efficiency and higher care quality when utilising telehealth in caring for older patients than in the regular in-person care mode.

**Conclusion:**

Since the beginning of the Covid-19 pandemic, telehealth has continued as an alternative platform in clinical services. However, as a healthcare platform that offers flexibilities of time, location, and improved efficiency, changing the traditional mindset is essential to shift the paradigm to use telehealth when appropriate. Importantly, telehealth needs substantial support in rural or remote long-term care facilities. Doing so will contribute to the reduction of healthcare inequity in long-term care facilities in remote settings and those with social disparities.

Telehealth and telecare will likely be part of the routine care delivery methods post the Covid-19 pandemic. A significant innovation, telehealth ensured timely care access during the pandemic in the general population as well as residents living in long-term care facilities (LTC). For the latter, this is particularly important and beneficial due to the high care demands of LTC residents, the limited workforce, and the unique environment of a long-term care facility where residents have limited mobility.

We previously reported a retrospective study of 27,980 residents aged 65 years or over in LTC settings (also known as residential aged care facilities in Australia) during the Covid-19 pandemic regarding the patterns of telephone or videoconference usage in general practice from March 2020 to August 2021 in New South Wales and Victoria in Australia. Furthermore, we identified residents’ socioeconomic characteristics of telehealth consultations to illustrate the barriers to using telehealth in the LTC setting [1].

Our comprehensive analyses show that telehealth visits significantly increased around the months when outbreaks occurred, ranging from the highest usage of 25% in August 2020 (strict lockdowns occurred during July—September in Victoria) to the lowest of 9% in April 2021 (schools and workplaces opened during November 2020 to May 2021). These data indicate that the telehealth utilisation rates correspond to the severity of the pandemic situations. However, over 90% of the telehealth visits were conducted via telephone, with residents who received government pensions (an indicator of low-income status) and rural residents (an indicator of living in rural or remote areas) being more likely to use telehealth (particularly phone) relative to face-to-face consultations, as compared to their counterparts. Additionally, video utilisation was evident with significantly lower usage than phone consultation in rural residents. These results were similar when stratified by different Covid surge, suggesting the consistency of findings of residents’ socioeconomic status in association with telehealth utilisation during the pandemic.

## Study implication

Our findings imply that social disparity during the Covid-19 pandemic further divided care access. Those who were financially sound or lived in urban areas were more likely to see a general practitioner (GP) in person. For example, when a facility is more equipped to control the spread of Covid infection, such as having sufficient care workers, protection measures, and equipment, residents are more likely to have more doctor’s visits than phone consultations. Moreover, the dire use of virtual care via videoconferencing indicates health disparity in information technology, such as slow or intermittent internet connection, lack of equipment, poor information and communication technology (ICT) skills, and low ICT literacy. These factors can significantly hamper the optimal use of virtual care in remote facilities, even though residents living in these areas would benefit the most from such technology-assisted care.

While our analysis provides comprehensive epidemiological data, it cannot unfold the voices of telehealth users, including patients, physicians, nurses, and care workers. Understanding these users’ perspectives would aid the improvement of care quality and efficiency in telehealth that centre on patients’ needs in LTC in an ongoing effort.

## Patients’ perspectives

It is no doubt that patients are at the centre of quality care. Among older patients aged 55 years and over, a study found that patients’ habits played the most critical role in using telehealth during the Covid-19 pandemic among various factors [2]. In addition, compared to patients in general, older adults residing in LTC often opt not to use virtual care due to the lack of skills in connecting to the internet or using digital devices [3]. Different reviews have mentioned that hearing, vision, or cognitive impairment can limit patients’ ability to use new technology to access care [3]. Furthermore, patients’ preferences for either phone or video consultations depend on the individuals and contexts. Before the pandemic, an Australian study found that patients aged 65 years and above expressed willingness to use telehealth services that offered all aspects of care, particularly among those living in remote areas with limited access to hospitals or clinics [4]. A U.S. study reported that the participating nursing homes preferred to continue using telemedicine post the Covid-19 pandemic, particularly in urgent assessments during residents’ acute change in conditions or cognitive-based sub-specialty consultations [5]. Similar suggestions were reported in a U.K. study that recommended the adoption of video consultations in settings such as nursing homes and remote areas due to video offering relatively more significant clinical advantages over phone consultations [6].

It should be noted that most studies on older patients’ preferences for phone or video visits were conducted in general clinical settings. While patients generally would be willing to use the video modality, older patients still prefer in-person visits over telehealth [7, 8]. For example, a U.S. nationally representative survey reported that 64.5% of the participants 60 years or older preferred in-person visits, and only 12.6% preferred video visits [7]. The Israeli study also reported that older patients of mean age of 61 were less likely to use telemedicine [8]. In another survey of patients in New Zealand, patients’ preferences for phone or video consultations depended on their condition or care requirement and their technical knowledge or skills to set up video calls [9]. Furthermore, patients and clinicians expressed concerns about the clinical effectiveness and the limitations of conducting physical examinations virtually [10]. Together, these factors contribute to the lower-than-optimal telehealth utilisation among LTC residents who are generally older.

## Physicians’ perspectives

The good news is that most healthcare practitioners have a positive attitude toward using telehealth [11–14]. Generally, physicians acknowledged that telehealth improved timely care access as well as care quality. However, a few major concerns were raised: 1) technical issues associated with equipment and 2) the expanded roles of LTC staff, with the latter increasing workloads that could affect clinical decision-making negatively and may even create medico-legal risks [13]. However, physicians also mentioned other benefits of using telehealth, such as reducing deferred care, improving physicians’ efficiency, and enhancing communication with caregivers and patients. Additionally, telehealth reduced travel burdens and facilitated health outreach and education [11]. 3) It is worth noting that unequal access is challenging for rural, older, and cognitively impaired patients [11]. Some physicians had concerns about taking physical examinations, picking up nonverbal cues, technological limitations, and maintaining doctor-patient relationships [14] when using telehealth.

It is expected that some physicians had not used telehealth until the Covid-19 pandemic when the government expanded telehealth broadly in the healthcare system due to the pandemic [12]. In line with this scenario, several studies reported that telephone consultation was the dominant mode during the Covid-19 pandemic, evident in our analyses [1] and others [15]. However, although physicians recognised the benefits of video over phone consultations, technological difficulties or technical skills to conduct video consultations remained the common barriers to adopting videoconferencing in  clinical care [16].

Interestingly, in a U.S. study among 814 clinicians of the Department of Veterans in New England, primary care clinicians (18.8%) and specialists (31.7%) had a similar preference for using telephone or video care during the Covid-19 pandemic. However, both raised the challenges of using video care in conducting a physical examination. By contrast, mental health clinicians (49.5%) preferred using videoconferencing over phone for new or existing patients remotely, and expressed higher quality and efficiency of using video in mental health consultations [17].

## Nurses’ and other healthcare workers’ perspectives

While most of the studies provided physicians' perspectives, understanding the perspectives of nurses and other healthcare workers is equally essential, as they are the backbone of the care workforce to ensure routine care schedules and logistics flow are on track.

In an Australian survey, twenty-five primary healthcare nurses acknowledged that telephone consultation was primarily used in care delivery during the first few months after the beginning of the pandemic in 2020. However, while most nurses and healthcare workers perceived telehealth positively as a way to enhance patients’ care access, the majority had concerns about acquiring appropriate knowledge of using the technology for themselves and patients [15].

On the other hand, health professional workers in LTC in France managing residents’ neuropsychiatric conditions perceived telehealth as a great platform to improve the quality of and access to care, particularly in remote areas [18]. These health workers also acknowledged cost-saving in healthcare, increased training opportunities for health professional workers, better communication with patients’ families, and reduced hospital transfers of residents in the LTC setting [18].

## Telehealth in rural or remote areas

Telehealth in rural or remote areas has a particular advantage in serving residents in LTC settings. A body of evidence before the Covid-19 pandemic has demonstrated the benefits of delivering care using telehealth in rural areas, particularly under conditions where healthcare professionals, services, and resources are limited. For example, nurses could handle a few more patients using telehealth than regular care when serving remote areas [19]. Another study also suggests that telehealth in nursing services slightly exceeded nurses’ expectations regarding the quality of care for older patients [20].

Perspectives from different users mentioned above illustrate that overcoming technology limitations and increasing user training are probably the most critical strategies to facilitate telehealth in rural or remote facilities. Decisions around phone versus video, the frequency and length of telehealth versus in-person consultation or care should be primarily based on patients’ clinical severity or  stability of the conditions, and the scheduling of physicians’ routine visits.

## Telehealth, a new normal

Since the beginning of the Covid-19 pandemic, telehealth has continued as an alternative platform in clinical services. Therefore, telehealth will likely stay as part of routine clinical services beyond the pandemic because of its advantages and benefits mentioned above [21, 22]. In LTC, telehealth offers efficiency and quality of care in managing routine medical check-ups, medication reviews, mental health consultations, nutrition, physiotherapy, and other allied health referrals, minor injuries, and chronic pain, among many chronic and common health conditions [23]. However, telehealth should not replace in-person care, especially in the circumstances such as conducting physical examinations, acute infections, and life-threatening events. Therefore, we should be mindful of weighing the nature and risk of the health conditions of LTC residents and balance telehealth utilisation with in-person care to tailor individual residents’ needs.

Additionally, in LTC settings, virtual platforms and internet technology can be introduced beyond clinical care, such as family/friend visits, group exercise, and entertainment, such as dancing and singing among residents. Indeed, some e-health services have already been available in commercial space [23]. A shift of mindset to adapt technology in clinical and routine care is probably the key to making such a paradigm in LTC settings [24].

## Conclusion

Telehealth offers flexibilities of time, location, and improved healthcare efficiency. However, its optimal utilisation in long-term care settings requires continuing government funding for suitable technology and devices, transparent billing codes, and policies protecting patients’ privacy and security. Importantly, telehealth needs substantial support in rural or remote long-term care facilities. Doing so will contribute to reducing healthcare inequity in remote settings and those with social disparities.

## Data Availability

Not applicable.
